# Observed RET-Positive Findings Across Routine Comprehensive Genomic Profiling Platforms in Japan: A Nationwide Descriptive Benchmark

**DOI:** 10.3390/cancers18111735

**Published:** 2026-05-26

**Authors:** Shinya Kajiura, Ryuji Hayashi

**Affiliations:** Department of Medical Oncology and Palliative Medicine, Toyama University Hospital, Toyama 930-0194, Japan; hsayaka@med.u-toyama.ac.jp

**Keywords:** RET fusion, comprehensive genomic profiling, precision oncology, real-world data, C-CAT, tumor-agnostic biomarker, cancer genomic medicine, liquid biopsy, platform heterogeneity

## Abstract

**Background:** RET fusion is an actionable tumor-agnostic biomarker, but its observed frequency in routine comprehensive genomic profiling (CGP) may vary across testing platforms and clinical contexts. We conducted a nationwide descriptive analysis to benchmark observed RET fusion frequency in Japanese routine practice. **Methods:** This retrospective descriptive study used anonymized aggregated data from the Center for Cancer Genomics and Advanced Therapeutics (C-CAT), including CGP-tested cases through 31 March 2025. Observed RET fusion frequency was summarized overall, across five standardized CGP platforms, across 12 prespecified organ groups, and in pooled tissue-based versus liquid-based comparisons. Exact binomial 95% confidence intervals were calculated to provide descriptive precision for low-frequency estimates. **Results:** Among 97,343 cases, 257 were RET-positive, corresponding to an overall observed RET fusion frequency of 0.26%. Platform-specific frequencies were 0.29% (192/66,992) for FoundationOne CDx, 0.28% (42/14,878) for FoundationOne Liquid CDx, 0.14% (6/4235) for GenMineTOP, 0.16% (15/9196) for NCC oncopanel, and 0.10% (2/2042) for Guardant360. Thoracic tumors showed the highest observed frequency (1.39%, 94/6740), followed by head and neck/thyroid tumors (1.04%, 42/4030). In a crude pooled comparison not adjusted for organ mix or clinical context, tissue-based and liquid-based CGP yielded numerically similar crude pooled frequencies of 0.265% (213/80,423) and 0.260% (44/16,920), respectively. **Conclusions:** This nationwide analysis benchmarks how RET-positive findings are surfaced to clinicians across heterogeneous routine CGP implementations in Japan. The data support platform-aware interpretation of RET results in practice, but should not be construed as biologic prevalence estimates or comparative assay performance.

## Simple Summary

RET fusion is a rare but clinically important biomarker because patients with RET-positive tumors may benefit from selective RET inhibitors. In Japan, the first reimbursed comprehensive genomic profiling (CGP) result can shape access to molecularly guided treatment, so clinicians need practical benchmarks for interpreting both positive and negative RET findings. In this nationwide analysis of 97,343 cases from the C-CAT cancer genomic medicine system, the overall observed RET frequency was 0.26%, with the highest frequencies in thoracic and head and neck/thyroid tumors. Observed frequencies differed across the five routinely used platforms, while a crude pooled tissue-based versus liquid-based comparison showed numerically similar crude pooled frequencies. These findings do not compare assay performance or prove interchangeability, but they provide a large real-world benchmark for platform-aware interpretation in routine precision oncology.

## 1. Introduction

RET fusions are clinically actionable oncogenic drivers that occur across multiple solid tumors, most prominently in lung and thyroid cancers but also in a broader range of gastrointestinal, breast, gynecologic, genitourinary, and other malignancies [1,2]. The development of selective RET inhibitors, particularly selpercatinib (Retevmo^®^, Eli Lilly and Company, Indianapolis, IN, USA) and pralsetinib, has expanded the clinical relevance of RET fusion beyond its canonical disease settings and reinforced its role as a tumor-agnostic biomarker in precision oncology [3,4,5,6,7]. In this context, understanding how RET fusion is observed in routine genomic testing is relevant not only for rare-biomarker epidemiology but also for real-world access to biomarker-directed treatment and for interpretation of actionable findings across tumor types [8,9]. Regulatory-review literature has also described the tissue-agnostic approval rationale for selpercatinib in advanced RET fusion-positive solid tumors [10]. In RET fusion-positive non-small cell lung cancer, first-line selective RET inhibition has been evaluated against platinum-based chemotherapy plus pembrolizumab [11]. In RET-activated thyroid cancer, long-term LIBRETTO-001 data have further supported durable responses in an established canonical RET-driven disease setting [12].

At the same time, RET fusion detection is not platform-neutral [1]. Gene fusion calling can be influenced by assay design, analyte type, specimen quality, and reporting architecture [13,14]. In particular, DNA-based comprehensive genomic profiling may face technical challenges when rearrangements involve large intronic regions or complex breakpoint structures, whereas assays that incorporate RNA interrogation may offer complementary advantages for identifying expressed fusion transcripts [3,15]. However, real-world testing environments are more complex than a simple DNA-versus-RNA dichotomy: tissue-based and liquid-based assays are used in different clinical contexts, circulating tumor DNA assays are affected by shedding and tumor fraction, and observed results can also reflect pre-analytic and reporting differences rather than intrinsic assay superiority [16,17,18].

These issues are especially relevant in Japan, where cancer genomic medicine has been implemented within a national framework and clinical-genomic data from routine comprehensive genomic profiling are aggregated through the Center for Cancer Genomics and Advanced Therapeutics (C-CAT) [19,20]. Because comprehensive genomic profiling is generally reimbursed once per patient in routine practice under the Japanese system, the result generated by a single test can carry substantial weight for biomarker interpretation, molecular tumor board deliberation, and potential treatment opportunity. Although nationwide C-CAT-based analyses have begun to describe rare actionable alterations in Japanese routine practice, large-scale descriptive data specifically showing how observed RET fusion frequency varies across routinely used comprehensive genomic profiling platforms remain limited [21,22,23]. Patient-reported experiences under the Japanese national health insurance system further underscore that implementation context matters for cancer genomic medicine [24]. The timing of CGP testing has also been studied as a practical determinant of how CGP information is used in advanced solid tumors in Japan [25]. Nationwide analyses of malignant solid tumors in Japan have provided broader genomic landscape data that contextualize rare-biomarker findings from routine CGP [26].

Against this background, we conducted a retrospective descriptive analysis of anonymized aggregated nationwide C-CAT data to characterize the observed RET fusion frequency across five routinely used comprehensive genomic profiling platforms and 12 prespecified organ groups in Japanese routine practice. Rather than re-estimating biologic prevalence or comparing assay performance, this study was designed to benchmark how RET-positive findings are surfaced to clinicians across heterogeneous routine CGP implementations in Japan, where reimbursed testing is generally performed once per patient, and thereby to support platform-aware interpretation of RET results in routine precision oncology.

## 2. Methods

### 2.1. Study Design and Data Source

This retrospective descriptive study used anonymized aggregated clinico-genomic data from the Center for Cancer Genomics and Advanced Therapeutics (C-CAT), the national data center for cancer genomic medicine in Japan. C-CAT aggregates clinical and genomic information generated through comprehensive genomic profiling (CGP) performed within the Japanese cancer genomic medicine system and therefore reflects routine nationwide clinical practice. The analytic dataset used for this study summarized aggregated nationwide C-CAT data available through 31 March 2025. Because the present study used anonymized aggregated data, the analysis was designed as an implementation-level descriptive assessment and did not permit patient-level linkage, longitudinal follow-up, or paired-sample comparisons across assays. The aggregated source dataset did not include case-level fusion-partner identities, fusion breakpoints, or actual post-CGP RET inhibitor treatment administration; therefore, these variables could not be analyzed.

### 2.2. Case Extraction and Study Framework

We conducted a retrospective nationwide descriptive analysis of CGP-tested cases represented in the aggregated C-CAT dataset. The analytic unit was the registered CGP-tested case as captured in the aggregated nationwide framework. Under the Japanese cancer genomic medicine system, CGP is generally reimbursed once per patient in routine practice; therefore, the nationwide C-CAT framework is expected to predominantly represent unique patients rather than repeated testing of the same individual, although this could not be formally verified at the patient level in the present aggregated dataset. The objective of the analysis was to describe the observed frequency of RET findings across routinely used CGP platforms, prespecified organ groups, and pooled specimen-context categories in Japanese routine practice rather than to re-estimate pan-cancer prevalence, evaluate assay performance, or perform paired concordance assessment. Consequently, tissue- and plasma-based results were not paired within the same patient, and any tissue/liquid comparison in this study should be read as a crude pooled implementation-level comparison rather than a concordance analysis.

### 2.3. Definition of RET Positivity

RET-positive cases were identified according to the operational positive class available in the source aggregated dataset. Specifically, a case was counted as positive when a rearrangement marker field contained RET and the corresponding change field was annotated as fusion, rearrangement, or rearrangements. Because these labels were grouped within the nationwide aggregated reporting framework available for secondary use, the present analysis describes this operational class as RET fusion findings for consistency across the manuscript, figures, and tables, while acknowledging that the individual structural nature of each RET-positive event could not be re-adjudicated. Accordingly, the analysis was intended to characterize how operationally defined RET-positive findings were observed in the aggregated routine-practice dataset rather than to adjudicate the exact structural class or biological validity of each individual event. Therefore, a small number of non-fusion rearrangements or structurally misclassified events, including intragenic deletions or copy-number-related events captured under aggregate rearrangement labels, cannot be excluded; such events could modestly inflate or deflate observed frequencies in platform-dependent ways. These data should therefore be interpreted as operational RET-positive findings rather than exact biologic RET fusion prevalence.

### 2.4. Organ-Group Classification

Cases were categorized into 12 prespecified organ groups: biliary tract, bowel, breast, esophagogastric, gynecologic, head and neck/thyroid, liver, central/peripheral nervous system, other, pancreas, thoracic, and genitourinary. The biliary tract group included Vater-related cases. The thoracic group included lung, thymus, and pleural tumors. The other group comprised several rare categories consolidated for this analysis, including adrenal tumors and other uncommon disease groups categorized outside the major organ-based classifications.

### 2.5. CGP Platform Classification

Platform names were standardized into five manuscript-level CGP categories: FoundationOne CDx, FoundationOne Liquid CDx, GenMineTOP, NCC oncopanel, and Guardant360. For pooled specimen-context analyses, platforms were additionally grouped as tissue-based CGP (FoundationOne CDx, GenMineTOP, and NCC oncopanel) and liquid-based CGP (FoundationOne Liquid CDx and Guardant360). These platform and pooled specimen-context classifications were used consistently across the overall, organ-group, and descriptive comparison frameworks.

### 2.6. Statistical Analysis

Observed frequency was calculated as the number of RET-positive cases divided by the total number of cases within each analytic category. Exact binomial 95% confidence intervals (Clopper–Pearson) were additionally calculated for the categories shown in Table 1 and Table S1 to provide descriptive precision for low-frequency estimates. Descriptive summaries were prepared overall, by CGP platform, by organ group, by organ-platform combination, and for pooled tissue-based versus liquid-based CGP. Given the aggregated descriptive design and the purpose of the current manuscript, no formal inferential hypothesis testing was incorporated into the present analysis.

### 2.7. Ethics and Informed Consent

This study used anonymized data derived from the C-CAT framework. Under the Japanese cancer genomic medicine system, patients provide informed consent before registration in C-CAT. The present secondary analysis used anonymized aggregated data obtained through the C-CAT data-use framework and was conducted in accordance with the relevant governance framework for secondary use of C-CAT data in Japan. The anonymized aggregated C-CAT data were accessed for research purposes on 29 January 2026. The authors did not have access to information that could identify individual participants during or after data collection. This secondary analysis was approved by the Institutional Review Board of Toyama University on 1 May 2024 (Approval No. R2024016) and the Information Utilization Review Board of C-CAT in August 2025 (Approval No. CDU2025-010N). The study was conducted in accordance with the Declaration of Helsinki, as revised in 2013.

## 3. Results

A total of 97,343 cases were included in this nationwide aggregated descriptive analysis across five standardized comprehensive genomic profiling (CGP) platforms and 12 organ groups. Overall, 257 operationally defined RET-positive cases were identified, corresponding to an observed RET fusion frequency of 0.26%. Table 1 summarizes the overall cohort, platform-specific frequencies, and organ-specific totals. The complete organ-by-platform matrix is provided in Supplementary Table S1.

**Figure 1:**
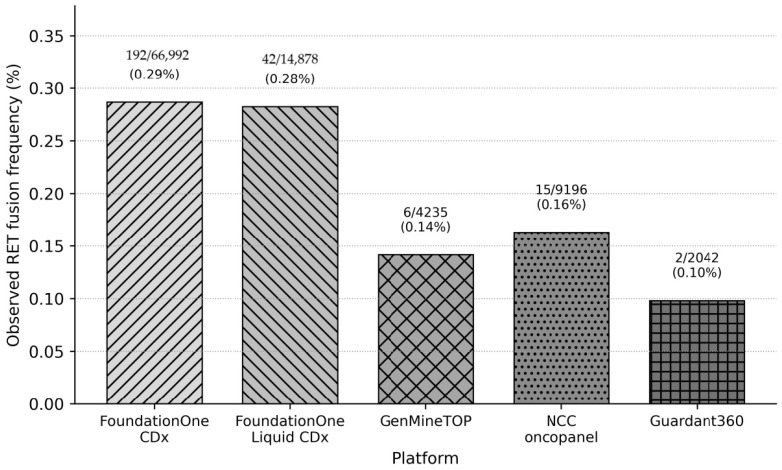
Overall observed RET fusion frequency by platform. Bars show the observed RET fusion frequency for each standardized comprehensive genomic profiling platform in the nationwide aggregated dataset. Observed frequency was calculated as RET-positive cases divided by total cases within each platform. Values above the bars indicate positive *n*/total *n* and the corresponding percentage. These data are descriptive and reflect cross-platform heterogeneity in this nationwide aggregated dataset; they should not be interpreted as a direct comparison of assay performance.

Overall observed RET fusion frequency varied across platforms (Figure 1; Table 1). Frequencies were 0.29% for FoundationOne CDx (192/66,992), 0.28% for FoundationOne Liquid CDx (42/14,878), 0.14% for GenMineTOP (6/4235), 0.16% for NCC oncopanel (15/9196), and 0.10% for Guardant360 (2/2042). Exact binomial 95% confidence intervals are shown in Table 1 to indicate descriptive precision for these low-frequency estimates. These findings indicate descriptive cross-platform heterogeneity in observed frequency within this nationwide aggregated dataset.

**Figure 2:**
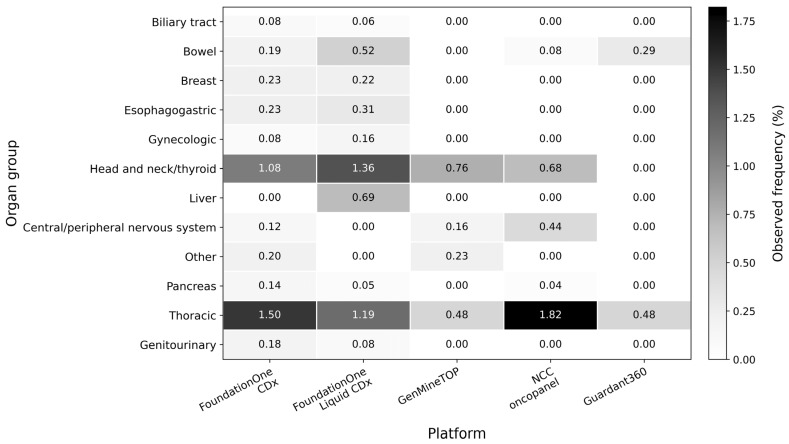
Organ-by-platform observed RET fusion frequency. The heatmap displays organ-specific observed RET fusion frequency (%) across the five standardized platforms. Each cell shows the percentage of RET-positive cases among all cases within that organ-platform category. The display is intended to visualize descriptive heterogeneity across organ groups and platforms in this nationwide aggregated dataset and should not be interpreted as evidence of platform superiority or inferiority. Cells with small denominators, including zero-event cells in very small categories such as Guardant360 in head and neck/thyroid (0/22) or liver (0/15), may appear visually distinct despite unstable estimates and should therefore be interpreted cautiously alongside Supplementary Table S1.

Across all platforms, observed RET fusion frequency was not uniform among organ groups (Table 1). Thoracic tumors showed the highest observed frequency at 1.39% (94/6740), followed by head and neck/thyroid tumors at 1.04% (42/4030). Frequencies in the remaining organ groups were lower, ranging from 0.07% in biliary tract and gynecologic tumors to 0.21% in bowel and esophagogastric tumors. These are broad organ-group estimates: the thoracic group includes lung, thymus, and pleural tumors, and the head and neck/thyroid group is a composite category rather than a thyroid-specific or head-and-neck-subtype-specific prevalence estimate. Accordingly, the frequencies shown in Table 1 and Figure 2 may smooth or obscure tumor-subtype-level variation and should be interpreted as descriptive grouped estimates.

Organ-by-platform variation was observed in the full matrix (Figure 2; Supplementary Table S1), although several cells were based on small denominators and should be interpreted cautiously. Thoracic tumors demonstrated RET-positive cases across all five platforms, with observed frequencies ranging from 0.48% to 1.82% depending on platform. Head and neck/thyroid tumors also showed positive cases across multiple platforms, with frequencies between 0.68% and 1.36% where cases were available. In several lower-frequency organ groups, positive cases were concentrated within selected platform-organ categories, whereas multiple sparse cells contained zero detected events. Among the 60 organ-platform cells excluding overall rows and columns, 28 cells had zero RET-positive events and 9 cells had denominators below 100, underscoring the need to interpret the heatmap as a descriptive visualization rather than as stable cell-level estimates.

**Figure 3:**
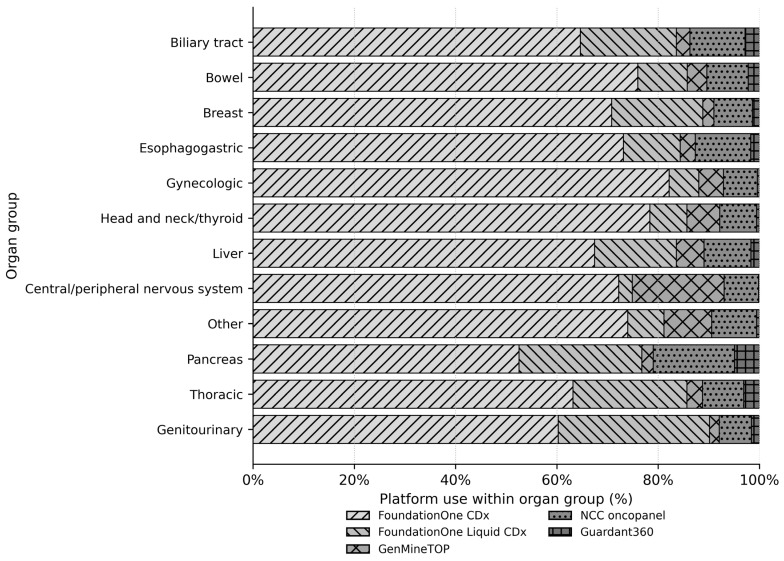
Platform-use distribution across organ groups. Horizontal stacked bars show the distribution of platform use within each organ group, based on the proportion of all cases assigned to each platform. This figure provides practice-context information to support interpretation of observed frequency heterogeneity by showing differences in platform mix across organ groups. Total cases by organ group were biliary tract (*n* = 9103), bowel (*n* = 15,791), breast (*n* = 7498), esophagogastric (*n* = 5823), gynecologic (*n* = 10,963), head and neck/thyroid (*n* = 4030), liver (*n* = 897), central/peripheral nervous system (*n* = 3357), other (*n* = 9369), pancreas (*n* = 15,270), thoracic (*n* = 6740), and genitourinary (*n* = 8502).

Platform-use distribution differed substantially by organ group (Figure 3). FoundationOne CDx accounted for the largest share of testing in most organ groups, whereas the relative use of FoundationOne Liquid CDx was greater in selected settings such as pancreas and genitourinary tumors. GenMineTOP and NCC oncopanel contributed variable proportions across organ groups, providing context for interpretation of observed organ-by-platform heterogeneity.

In a crude pooled comparison not adjusted for organ mix or clinical context, tissue-based CGP platforms (FoundationOne CDx, GenMineTOP, and NCC oncopanel) yielded 213 RET-positive cases among 80,423 tested cases (0.265%), whereas liquid-based CGP platforms (FoundationOne Liquid CDx and Guardant360) yielded 44 RET-positive cases among 16,920 tested cases (0.260%) (Supplementary Figure S1). The crude pooled frequencies were therefore numerically similar in this descriptive aggregated dataset. This pooled comparison was descriptive and unadjusted, and should therefore be interpreted cautiously because it did not account for organ mix, clinical context, or sample context and because the liquid-based pool was dominated by FoundationOne Liquid CDx.

## 4. Discussion

In this nationwide aggregated descriptive analysis of 97,343 comprehensive genomic profiling (CGP)-tested cases, operationally defined RET-positive findings were infrequent overall but showed clear concentration in thoracic and head and neck/thyroid tumors and descriptive heterogeneity across the five standardized platforms. The central implication of these findings is that observed RET positivity in routine CGP reflects not only tumor biology but also testing context, platform use, case selection, and aggregation rules. Thus, the value of this nationwide benchmark lies in helping clinicians and molecular tumor boards interpret operational RET-positive and RET-negative findings within real-world implementation constraints rather than treating observed frequencies as biologic prevalence estimates or comparative assay-performance results.

The most notable interpretive point is that crude observed frequencies in this nationwide aggregated dataset did not align with a simple expectation that RNA-supportive testing settings would necessarily yield higher RET positivity. This descriptive pattern should not be interpreted as evidence regarding analytical sensitivity, assay hierarchy, or equivalence among platforms. Prior literature has emphasized that RNA interrogation can offer important advantages for detecting expressed fusion transcripts, particularly when DNA breakpoints are complex, intronic, or otherwise challenging to resolve [3,15,27]. However, the present study was not designed as a paired-sample comparison or assay-validation study. Rather, it captures implementation-level observed frequency in routine care, which reflects a composite of biology, specimen context, assay design, reporting rules, and case selection rather than intrinsic platform performance.

Several non-mutually exclusive mechanisms may explain why crude real-world observations did not map neatly onto a simple analytic expectation about RNA-favored fusion detection. First, pre-analytic conditions matter greatly. RNA is biologically vulnerable and can be disproportionately affected by fixation, ischemic time, archival age, tissue adequacy, and nucleic-acid degradation, so any potential benefit of RNA interrogation may be attenuated in routine specimens that are less than ideal [3,27]. Second, platform differences extend beyond a simple DNA-versus-RNA distinction and may include targeted genomic regions, assay architecture, reporting logic, and thresholds for calling clinically reportable events [3,28]. Third, routine platform selection is not random. As shown by the platform-use distribution across organ groups, different assays are preferentially used in different tumor contexts, with differing specimen availability, disease settings, and institutional workflows. Accordingly, apparent cross-platform heterogeneity in observed frequency may reflect case mix and implementation pathways as much as, or more than, analytic factors alone [19,22,29]. The practical contribution of the present study is therefore not to identify the “best” assay, but to provide a practice-facing interpretive benchmark for how RET-positive findings emerge under heterogeneous routine testing conditions. Accordingly, the observed platform-specific frequencies should be regarded as implementation-level observed frequencies shaped by analyte type, specimen quality, tumor type, specimen availability, institutional workflow, and non-random assay selection, not as direct measures of assay sensitivity, assay superiority, or platform ranking.

This issue has particular clinical meaning in Japan. Within the Japanese cancer genomic medicine framework, CGP is generally reimbursed once per patient in routine practice, and the result generated by that first test can substantially influence subsequent molecular tumor board discussion, referral decisions, and treatment opportunity [19,20,21,29]. For a rare but actionable biomarker such as RET fusion, uncertainty in interpretation therefore has concrete downstream implications. A negative RET result obtained on one platform should not automatically be understood as definitive biological absence, especially when clinicopathologic features raise suspicion for a RET-driven tumor. Rather, the negative result must be interpreted in light of specimen type, sample quality, platform context, and the broader clinical picture. In that sense, our data reinforce a platform-aware approach to molecular interpretation in routine precision oncology rather than a binary positive-versus-negative reading detached from testing context. In this setting, a RET-positive result can influence molecular tumor board discussion and referral decisions, whereas a RET-negative result may affect whether clinicians consider additional tissue acquisition, alternative testing, or clinical-trial referral when clinically appropriate and available.

The numerically similar crude pooled frequencies between tissue-based CGP and liquid-based CGP also merit careful interpretation. These data do not establish equivalence between tissue and plasma testing, nor do they imply paired concordance at the patient level. Rather, they represent a crude implementation-level observation derived from aggregated data in which organ mix, disease stage, circulating tumor DNA shedding, sampling timing, tumor burden, and the clinical reasons for selecting liquid biopsy were not standardized. Accordingly, this finding should not be interpreted as evidence that tissue and plasma testing are interchangeable for RET fusion detection. At the same time, liquid biopsy remains clinically relevant as part of the biomarker-detection ecosystem for advanced cancer, particularly when tissue is limited or repeat biopsy is infeasible [16,17]. Fusion detection in plasma is strongly influenced by circulating tumor DNA shedding, tumor fraction, disease burden, timing of sampling, and treatment context, and therefore a liquid-negative result cannot be interpreted in the same way across all patients [16,17,18]. Our findings thus support cautious clinical use of liquid-based CGP for RET interpretation while preserving the need for context-aware judgment rather than assuming interchangeability with tissue testing. International recommendations likewise emphasize that ctDNA assay results should be interpreted in the context of tumor fraction, assay limitations, and the clinical scenario [30]. In the present dataset, however, FoundationOne Liquid CDx accounted for most liquid-based testing, so the pooled liquid estimate should be understood primarily as a FoundationOne Liquid CDx-weighted observation and not as a platform-neutral estimate for liquid-based CGP in general. Guardant360 CDx has been clinically validated as a blood-based companion diagnostic platform in advanced NSCLC [31]. The same caution applies to negative liquid results: absence of a reported RET alteration in plasma may reflect low tumor fraction, limited shedding, sampling timing, or platform context, and should not be converted into a definitive biological exclusion when the clinical scenario remains suspicious.

The organ distribution observed in the present study is also biologically and clinically coherent. The concentration of RET-positive cases in thoracic tumors and head and neck/thyroid tumors aligns with the established disease contexts in which RET fusions are most often recognized, especially non-small cell lung cancer and thyroid malignancies [3,8,9,32]. At the same time, the presence of RET-positive cases across a broader range of organ groups is consistent with the growing understanding of RET fusion as a tumor-agnostic actionable alteration, even when the absolute frequency within any given noncanonical tumor type is low [4,5,7]. In the current therapeutic era, such low-frequency events remain clinically meaningful because each detected case may create access to a highly relevant molecularly matched option. For this reason, large-scale descriptive benchmarking has value even when the headline frequency is numerically small. Conversely, lower absolute frequencies in gastrointestinal and other noncanonical organ groups should not be interpreted as absence of clinical relevance, because rare actionable fusions may still have important consequences for individual patients. Because these organ groups are broad composites, the values should not be read as tumor-subtype-specific prevalence estimates.

More broadly, our findings extend the emerging Japanese literature on routine CGP implementation and nationwide molecular epidemiology. Prior work has established the structure of C-CAT and the broader national cancer genomic medicine framework, and institutional reports have shown that real-world CGP results can translate into genomically matched treatment for a subset of patients [19,21,29]. Nationwide rare-biomarker analyses have also illustrated how large Japanese CGP datasets can clarify the real-world distribution of uncommon actionable alterations across tumor types [23]. Against that background, the present study contributes RET-specific evidence by focusing not only on pan-cancer occurrence but also on platform context. This is relevant beyond Japan, because many oncology systems increasingly rely on multiple CGP assays, both tissue-based and liquid-based, with heterogeneous implementation pathways. A nationwide benchmark of observed RET fusion frequency may therefore help readers interpret how assay context, specimen context, and clinical context intersect when an actionable fusion is sought in routine practice [22,33]. For international readers, therefore, the transferable message is not the exact Japanese frequency value, but the need to interpret actionable fusion findings through the lens of specimen choice, platform architecture, reporting conventions, and local implementation pathways.

This study has several important limitations. First, it was based on anonymized aggregated data and therefore did not allow patient-level linkage, review of individual clinicopathologic variables, or identification of repeat testing at the individual level. Second, the aggregate, non-paired design precluded concordance analyses or direct within-patient platform-performance comparisons. Third, the observed frequencies reported here cannot be disentangled from the underlying biological frequency of RET fusion, because platform selection in routine care was non-random and likely influenced by tumor type, specimen availability, institutional preference, disease status, and other clinical factors. Fourth, we lacked specimen-level pre-analytic information such as fixation conditions, tumor fraction, nucleic-acid quality, metastatic burden, timing of sampling, prior therapy, and fusion-partner details, all of which may influence observed detection. Fifth, treatment and outcome data were not incorporated into the present descriptive analysis, so the downstream clinical utility of observed RET positivity could not be directly assessed. Sixth, several organ-platform cells were sparse, and zero-event or very low-count cells may be unstable and should not be overinterpreted. Seventh, some broad organ groups, particularly head and neck/thyroid and thoracic, combine biologically heterogeneous tumor types and may therefore smooth tumor-specific RET patterns within composite categories. Finally, as defined in Methods, RET positivity was based on an operational aggregate class, and this approach may include some non-fusion rearrangements or structurally misclassified events; therefore, the observed frequencies should not be interpreted as exact biologic RET fusion prevalence. In addition, broad organ-group aggregation, particularly for thoracic and head and neck/thyroid tumors, precludes tumor-subtype-specific prevalence estimation. This limitation profile also underscores the importance of transparent reporting in studies conducted using routinely collected health data [34]. These constraints also mean that neither a platform-specific positive rate nor a negative test result should be treated as standalone evidence of assay performance, definitive biological absence, prognosis, treatment eligibility, treatment receipt, or clinical benefit. In addition, because case-level fusion-partner identities, fusion breakpoints, and actual post-CGP RET inhibitor administration were unavailable, a fusion-partner table by cancer type and an assessment of RET inhibitor uptake, response, survival, or clinical benefit could not be generated from the current source dataset.

## 5. Conclusions

Despite these limitations, a nationwide descriptive analysis of nearly 100,000 CGP-tested cases offers a scale of evidence that is difficult to obtain in single-center series. In conclusion, RET fusion findings were infrequent overall but showed clear concentration in thoracic and head and neck/thyroid tumor contexts and clear heterogeneity across routinely used CGP platforms in Japan. The fact that crude observed frequencies did not map neatly onto a simple analytic expectation about RNA-supportive settings underscores that routine RET interpretation must remain platform-aware and context-aware rather than reductionist. As a nationwide real-world benchmark, this study may assist oncologists, molecular tumor boards, and translational researchers in interpreting both positive and negative operationally defined RET findings more carefully in everyday precision oncology.

## Figures and Tables

**Table 1:** Overall observed RET fusion frequency in the nationwide aggregated dataset and organ-specific totals. Panel A summarizes the overall total and overall platform-specific frequencies. Panel B summarizes organ-specific totals across all platforms. The complete organ-by-platform matrix is provided in Supplementary Table S1 for readability. Observed frequency was calculated as RET-positive cases divided by total cases in each category, and exact binomial 95% confidence intervals are shown to indicate descriptive precision. These data reflect descriptive cross-platform heterogeneity in this nationwide aggregated dataset and should not be interpreted as a direct assay-performance comparison.

**Panel A. Overall and Platform-Specific Frequencies**	
**Category**	**RET-Positive/Total (%)**
Overall (all platforms)	257/97343 (0.26%) 95% CI, 0.23–0.30%
FoundationOne CDx	192/66992 (0.29%) 95% CI, 0.25–0.33%
FoundationOne Liquid CDx	42/14878 (0.28%) 95% CI, 0.20–0.38%
GenMineTOP	6/4235 (0.14%) 95% CI, 0.05–0.31%
NCC oncopanel	15/9196 (0.16%) 95% CI, 0.09–0.27%
Guardant360	2/2042 (0.10%) 95% CI, 0.01–0.35%
**Panel B. Organ-Specific Totals Across All Platforms**	
**Organ Group**	**RET-Positive/Total (%)**
Biliary tract	6/9103 (0.07%) 95% CI, 0.02–0.14%
Bowel	33/15791 (0.21%) 95% CI, 0.14–0.29%
Breast	15/7498 (0.20%) 95% CI, 0.11–0.33%
Esophagogastric	12/5823 (0.21%) 95% CI, 0.11–0.36%
Gynecologic	8/10963 (0.07%) 95% CI, 0.03–0.14%
Head and neck/thyroid	42/4030 (1.04%) 95% CI, 0.75–1.41%
Liver	1/897 (0.11%) 95% CI, 0.00–0.62%
Central/peripheral nervous system	5/3357 (0.15%) 95% CI, 0.05–0.35%
Other	16/9369 (0.17%) 95% CI, 0.10–0.28%
Pancreas	14/15270 (0.09%) 95% CI, 0.05–0.15%
Thoracic	94/6740 (1.39%) 95% CI, 1.13–1.70%
Genitourinary	11/8502 (0.13%) 95% CI, 0.06–0.23%

## Data Availability

The authors do not control the underlying C-CAT database. The data used in this study were obtained as anonymized aggregated counts through the approved C-CAT data-use framework and are subject to institutional and data-provider governance restrictions. Therefore, the underlying source-level C-CAT data are not publicly available from the authors. Access to source-level C-CAT data requires formal application to C-CAT and the relevant institutional approvals under the Japanese cancer genomic medicine governance framework.

## Supplementary Materials

The following supporting information can be downloaded at https://www.mdpi.com/article/10.3390/cancers18111735/s1: Figure S1: Pooled tissue-based versus liquid-based observed RET fusion frequency; Table S1: Observed RET fusion frequencies by organ group and platform.
