# Supplementary material for: Observed RET-Positive Findings Across Routine Comprehensive Genomic Profiling Platforms in Japan: A Nationwide Descriptive Benchmark
Source: Cancers (Basel). 2026 May 26;18(11):1735. doi: 10.3390/cancers18111735 (PMC13255864; doi:10.3390/cancers18111735)
Supplement: Supplementary file 1 [file cancers-18-01735-s001.zip › cancers-4289343-supplementary.pdf]

Supplementary Material: Observed RET-Positive Findings Across Routine Comprehensive Genomic Profiling Platforms in Japan: A Nationwide Descriptive Benchmark

Shinya Kajiura and Ryuji Hayashi

Table S1. Observed RET fusion frequencies by organ group and platform. Each cell shows positive *n*/total *n* (%) with exact binomial 95% confidence intervals. The Overall row summarizes the full nationwide aggregated dataset. Observed frequency was calculated as RET-positive cases divided by total cases in each category. These data are descriptive and should not be interpreted as a direct comparison of assay performance. Cells with small denominators should be interpreted cautiously.

Organ group	Overall	FoundationOne CDx	FoundationOne Liquid CDx	GenMineTOP	NCC oncopanel	Guardant360
Overall	257/97,343 0.26% (0.23–0.30)	192/66,992 0.29% (0.25–0.33)	42/14,878 0.28% (0.20–0.38)	6/4235 0.14% (0.05–0.31)	15/9196 0.16% (0.09–0.27)	2/2042 0.10% (0.01–0.35)
Biliary tract	6/9103 0.07% (0.02–0.14)	5/5884 0.08% (0.03–0.20)	1/1727 0.06% (0.00–0.32)	0/243 0.00% (0.00–1.51)	0/990 0.00% (0.00–0.37)	0/259 0.00% (0.00–1.41)
Bowel	33/15,791 0.21% (0.14–0.29)	23/12,000 0.19% (0.12–0.29)	8/1543 0.52% (0.22–1.02)	0/610 0.00% (0.00–0.60)	1/1288 0.08% (0.00–0.43)	1/350 0.29% (0.01–1.58)
Breast	15/7498 0.20% (0.11–0.33)	12/5311 0.23% (0.12–0.39)	3/1349 0.22% (0.05–0.65)	0/164 0.00% (0.00–2.22)	0/571 0.00% (0.00–0.64)	0/103 0.00% (0.00–3.52)
Esophagogastric	12/5823 0.21% (0.11–0.36)	10/4259 0.23% (0.11–0.43)	2/654 0.31% (0.04–1.10)	0/173 0.00% (0.00–2.11)	0/638 0.00% (0.00–0.58)	0/99 0.00% (0.00–3.66)
Gynecologic	8/10,963 0.07% (0.03–0.14)	7/9008 0.08% (0.03–0.16)	1/643 0.16% (0.00–0.86)	0/534 0.00% (0.00–0.69)	0/734 0.00% (0.00–0.50)	0/44 0.00% (0.00–8.04)
Head and neck/thyroid	42/4030 1.04% (0.75–1.41)	34/3159 1.08% (0.75–1.50)	4/294 1.36% (0.37–3.45)	2/262 0.76% (0.09–2.73)	2/293 0.68% (0.08–2.44)	0/22 0.00% (0.00–15.44)
Liver	1/897 0.11% (0.00–0.62)	0/605 0.00% (0.00–0.61)	1/145 0.69% (0.02–3.78)	0/49 0.00% (0.00–7.25)	0/83 0.00% (0.00–4.35)	0/15 0.00% (0.00–21.80)
Central/peripheral nervous system	5/3357 0.15% (0.05–0.35)	3/2425 0.12% (0.03–0.36)	0/90 0.00% (0.00–4.02)	1/608 0.16% (0.00–0.91)	1/229 0.44% (0.01–2.41)	0/5 0.00% (0.00–52.18)
Other	16/9369 0.17% (0.10–0.28)	14/6935 0.20% (0.11–0.34)	0/670 0.00% (0.00–0.55)	2/881 0.23% (0.03–0.82)	0/833 0.00% (0.00–0.44)	0/50 0.00% (0.00–7.11)

Organ group	Overall	FoundationOne CDx	FoundationOne Liquid CDx	GenMineTOP	NCC oncopanel	Guardant360
Pancreas	14/15,270<br>0.09%<br>(0.05–0.15)	11/8021<br>0.14%<br>(0.07–0.25)	2/3707<br>0.05%<br>(0.01–0.19)	0/340<br>0.00%<br>(0.00–1.08)	1/2450<br>0.04%<br>(0.00–0.23)	0/752<br>0.00%<br>(0.00–0.49)
Thoracic	94/6740<br>1.39%<br>(1.13–1.70)	64/4260<br>1.50%<br>(1.16–1.91)	18/1516<br>1.19%<br>(0.71–1.87)	1/207<br>0.48%<br>(0.01–2.66)	10/549<br>1.82%<br>(0.88–3.32)	1/208<br>0.48%<br>(0.01–2.65)
Genitourinary	11/8502<br>0.13%<br>(0.06–0.23)	9/5125<br>0.18%<br>(0.08–0.33)	2/2540<br>0.08%<br>(0.01–0.28)	0/164<br>0.00%<br>(0.00–2.22)	0/538<br>0.00%<br>(0.00–0.68)	0/135<br>0.00%<br>(0.00–2.70)

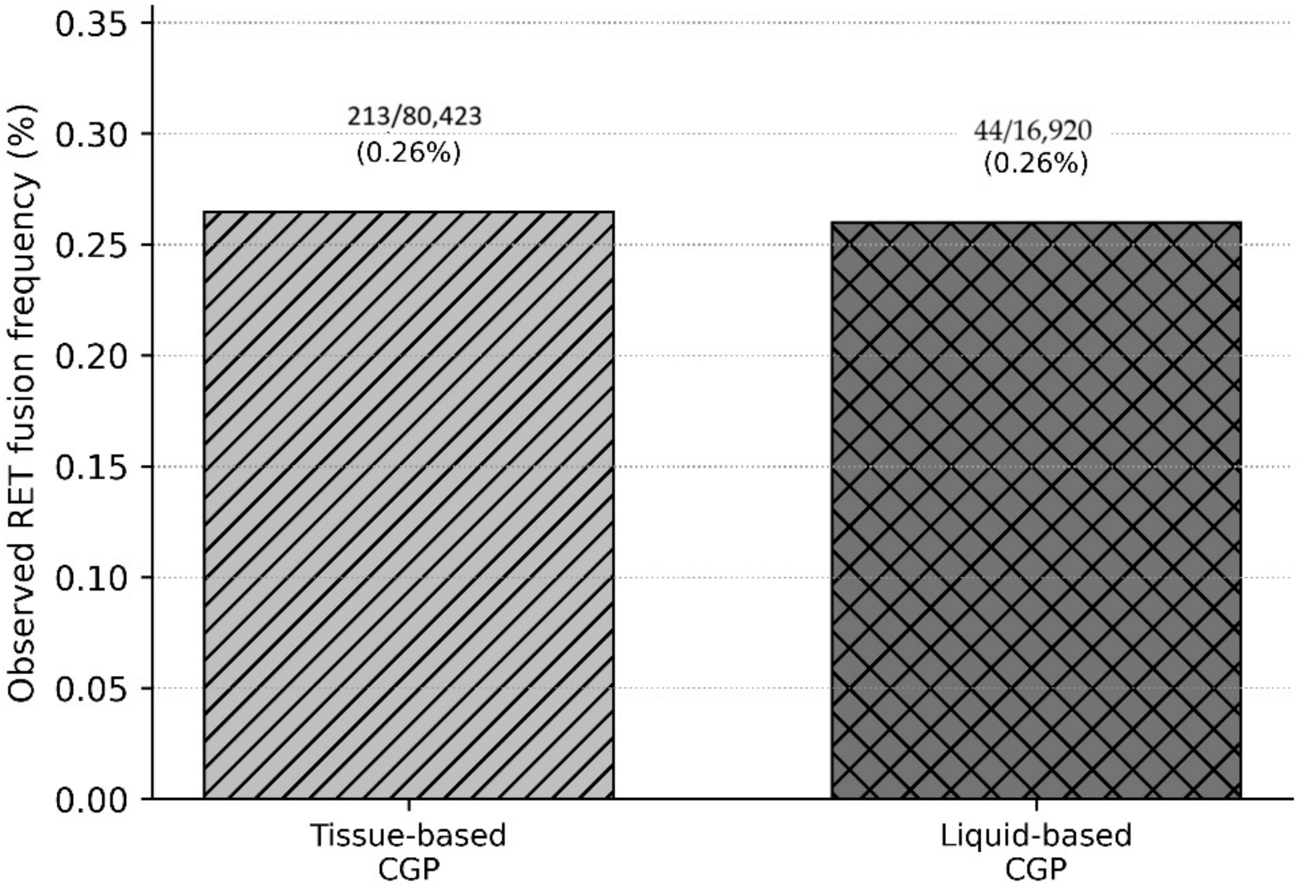

Figure S1. Pooled tissue-based versus liquid-based observed RET fusion frequency. Tissue-based comprehensive genomic profiling (CGP) was defined as FoundationOne CDx, GenMineTOP, and NCC oncopanel. Liquid-based CGP was defined as FoundationOne Liquid CDx and Guardant360. Observed frequency was calculated as RET-positive cases divided by total cases in each pooled category. This pooled comparison is crude and not adjusted for organ mix or clinical context; it should not be interpreted as evidence of paired concordance, assay equivalence, or interchangeability between tissue and plasma testing.
